# Medical Society of the County of Erie, January 13, 1908

**Published:** 1908-03

**Authors:** F. C. Gram


### SOCIETY PROCEEDINGS.

### Medical Society of the County of Erie, January 13, 1908.

Synopsis furnished by F. C. GRAM, M.D., Secretary.

THE regular meeting of the Medical Society of the County of Erie was held in the rooms of the Buffalo Society of Natural Sciences on January 13, 1908, at 4.30 P. M., the president, Dr. Albert H. Briggs, in the chair.

After approving the minutes of the October meeting and the meeting of the Council, the following names were presented by the Committee on Membership, and the secretary was instructed to cast the ballot of the society for their election:

Charles H. Andrews, 588 W. Delavan Avenue, Buffalo.
Christian L. Suess, 75 Central Avenue, Lancaster, N. Y.
Ralph Robinson, 910 Ridge Road, West Seneca, N. Y.
Edwin A. Bowerman, 234 E. Utica Street, Buffalo.
Chas. Williams Bethune, 904 Tonawanda Street, Buffalo.
Lesser Kauffman, 991 William Street, Buffalo.
Eugene O. Bardwell, 1175 Main Street, Buffalo.

Dr. Henry R. Hopkins, Chairman of the Board of Censors, then read a report in which he explained, in detail, the work done by the Board of Censors during the past year, especially so much as related to the cases of W. W. Turver and H. J. Ince, both of whom were convicted, and sentenced to prison, and whose licenses have since been revoked as a result of such conviction and imprisonment.

He also referred to the Board of Censors' action in the case of E. L. Schwabe who was prevented from taking an examination before the State Examining Board, but which action was afterward reversed by the Regents, thereby permitting him to take such examination.

This report elicited a warm discussion by many of the members present. It was finally adopted by a vote of 28 to 8.

Dr. Wall asked unanimous consent for the suspension of the by-laws, and moved that the dues for each member during the year 1908 be $2.00.

Motion was carried.

Dr. Wall also asked unanimous consent to suspend Section 1 of Chapter 15 of the by-laws which was granted. He then moved the following amendment to the by-laws which was unanimously adopted:

Amendment to By-laws:

Chapter IX, Sec. 1. Strike out "from the first of the following January" and add "they shall assume office at the close of the annual meeting."

Chapter XI, Sec. 1. Amend to read "Regular meetings shall be held on the third Monday in the months of February, April, June, October and December, as arranged by the Council.

"The December meeting shall be the Annual Meeting."

Sec. 2. Substitute (5) five for (10) ten.

Chapter XII. Sec. 1. Substitute "May" for "March."

On motion of Dr. Rochester the society instructed its delegates in favor of Dr. Charles G. Stockton for president of the Medical Society of the State of New York.

Dr. A. H. Briggs, the retiring president, then introduced Dr. Edward Clark as the president of the society for 1908.

Dr. Clark, in assuming his duties, briefly thanked the society, after which adjournment followed.

